# Mitochondrial dysfunction and cell senescence — skin deep into mammalian aging

**DOI:** 10.18632/aging.100432

**Published:** 2012-02-12

**Authors:** Joao F. Passos, Thomas von Zglinicki

**Affiliations:** Institute for Ageing and Health, Newcastle University, Newcastle upon Tyne, NE4 5PL, UK

There is a lively discussion going on as to whether oxidative stress is or is not a cause of (accelerated) aging, fuelled to a significant extent by the finding from Arlan Richardson's group that mice heterozygous for the mitochondrial superoxide dismutase SOD2 showed increased oxidative stress, increased cancer incidence but not accelerated ageing [1]. A new twist to this story was introduced recently when it was shown that connective tissue-specific SOD2 knockouts developed multiple signs of progeria including short lifespan, associated with up-regulation of the cell senescence marker p16^INK4A^ [2]. Mitochondrially generated oxidative stress is both an established cause [3] and a relevant consequence [4] of cell senescence, frequencies of senescent cells in connective tissue increase during mice aging [5], and destruction of senescent cells can ‘cure’ some age-related tissue dysfunction [6]. A paper by Judith Campisi's and Simon Melov's groups recently published in Aging [7] now further explores the connection between oxidative stress, cell senescence and aging. The authors demonstrate that mitochondrial dysfunction occurs in the epidermis of old (2 years) mice, measured as decreased complex II activity, and correlate this with increased senescence (shown by SA-bGAL activity) in the stratum corneum. Moreover, they observe the same senescence phenotype in skin from young (17 – 20 days old) constitutive SOD2−/− mice, which were treated with the synthetic SOD and catalase mimetic EUK-189 in order to allow sufficient development to take place for a skin phenotype to develop. An increase of various senescence markers in the epidermis, the stratum corneum or the lining of the hair follicles was associated with epidermal thinning (a classical aging marker in skin) and increased expression of a keratinocyte terminal differentiation marker [7]. These data enforce two central hypotheses in the field, namely that of mitochondrial dysfunction as a cause of cell senescence, and of cell senescence as a relevant contributor to mammalian aging *in vivo*.

However, a fascinating question remains: Is it really Reactive Oxygen Species (ROS) arising from mitochondria that promote cellular senescence in this model? Mitochondria from SOD2−/− mice accumulate superoxide in their matrix space which oxidizes and damages multiple mitochondrial enzyme complexes, leading to decreased oxygen uptake and ATP production and lowered complex II activity [8, 9]. However, superoxide cannot cross the mitochondrial inner membrane, and the generation of hydrogen peroxide, which is the only membrane-permeable ROS, is greatly reduced by two factors, the absence of the mitochondrial SOD and the decrease in oxygen uptake. Thus, SOD2−/− mitochondria actually release less ROS than wild-type mitochondria into their environment^9^. This is attenuated by the addition of the superoxide mimetic EUK-189, but even under high drug concentrations the ROS release from heart mitochondria was below wild-type levels [9]. Thus, it is not at all clear that mitochondrial oxidative stress directly produces cellular oxidative stress in the skin of EUK-189 treated SOD2−/− mice. Importantly, studies have shown that mitochondrial dysfunction in SOD2−/− fibroblasts is associated with Ca^2+^ signal transduction, suppression of signals through the mTOR axis and induction of markers of autophagy [8]. All these changes are characteristically associated with cell senescence [3, 10, 11]. Thus, we are again left with a chicken-and egg situation: Is disrupted Ca^2+^ signalling, reduced mTOR activity and increased autophagy found in SOD2−/− cells because there are more senescent cells in the examined population? Or is any of these factors the culprit that triggers senescence in the first place?

While the answer to these questions still eludes us, the study from Campisi and colleagues highlights the importance of mitochondrial dysfunction and cellular senescence in vivo and its impact on the aging process.

## References

[R1] Van Remmen H, Ikeno Y, Hamilton M (2003). Physiol Genomics.

[R2] Treiber N, Maity P, Singh K Aging Cell.

[R3] Passos JF, Saretzki G, Ahmed S (2007). PLoS Biol.

[R4] Passos JF, Nelson G, Wang C (2010). Mol Syst Biol.

[R5] Wang C, Jurk D, Maddick M (2009). Aging Cell.

[R6] Baker DJ, Wijshake T, Tchkonia T

[R7] Velarde MC, Flynn JM, Day NU (2012). Aging (Albany NY).

[R8] Zhang Y, Zhang HM, Shi Y (2010). Free Radical Biology and Medicine.

[R9] Morten KJ, Ackrell BA, Melov S (2006). J Biol Chem.

[R10] Korotchkina LG, Leontieva OV, Bukreeva EI (2010). Aging (Albany NY).

[R11] Sitte N, Merker K, Grune T (2001). Experimental Gerontology.

